# Factors associated with incomplete childhood immunization in Arbegona district, southern Ethiopia: a case – control study

**DOI:** 10.1186/s12889-015-2678-1

**Published:** 2016-01-12

**Authors:** Abel Negussie, Wondewosen Kassahun, Sahilu Assegid, Ada K. Hagan

**Affiliations:** 1 Department of Social and Population Health, Yirgalem Medical Science College, Yirgalem, Ethiopia; 2 Department of Epidemiology and Biostatistics, College of Public Health and Medicine, Jimma University, Jimma, Ethiopia; 3 Department of Microbiology and Immunology, Hanna Lab, University of Michigan Medical School, Ann Harbor, USA

**Keywords:** Incomplete immunization, Case–control study, Southern Ethiopia

## Abstract

**Background:**

The prevention of child mortality through immunization is one of the most cost-effective and widely applied public health interventions. In Ethiopia, the Expanded Program on Immunization (EPI) schedule is rarely completed as planned and the full immunization rate is only 24 %. The objective of this study was to identify determinant factors of incomplete childhood immunization in Arbegona district, Sidama zone, southern Ethiopia.

**Methods:**

A community based unmatched case-control study was undertaken among randomly selected children aged 12 to 23 months and with a total sample size of 548 (183 cases and 365 controls). A multi-stage sampling technique was used to get representative cases and controls. Data was collected using a structured questionnaire and analyzed using SPSS version 16 statistical software. Bivariate and multiple logistic regression analyses were done to identify independent factors for incomplete immunization status of children. Qualitative data were also generated and analyzed using thematic framework.

**Results:**

The incomplete immunization status of children was significantly associated with young mothers (AOR = 9.54; 95 % CI = 5.03, 18.09), being born second to fourth (AOR = 3.64; 95 % CI = 1.63, 8.14) and being born fifth or later in the family (AOR = 5.27; 95 % CI = 2.20, 12.64) as compared to being born first, a mother’s lack of knowledge about immunization benefits (AOR = 5.51; 95 % CI = 1.52, 19.94) and a mother’s negative perception of vaccine side effects (AOR = 1.92; 95 % CI = 1.01, 3.70). The qualitative finding revealed that the migration of mothers and unavailability of vaccines on appointed immunization dates were the major reasons for partial immunization of children.

**Conclusion:**

To reduce the number of children with incomplete immunization status, the Arbegona district needs to consider specific planning for mothers with these risk profiles. A focus on strengthening health communication activities to raise immunization awareness and address concerns of vaccine side effects at community level is also needed. This could be achieved through integrating the immunization service to other elements of primary health care.

**Electronic supplementary material:**

The online version of this article (doi:10.1186/s12889-015-2678-1) contains supplementary material, which is available to authorized users.

## Background

The prevention of child mortality through immunization is one of the most cost-effective and widely applied public health interventions. The Expanded Program on Immunization (EPI) aims at delivering the primary immunization series to at least 90 % of infants. However, the goal is still not achieved by many developing countries [1, 2]. Globally, approximately 21.8 million eligible children did not receive 3 doses of diphtheria-tetanus-pertussis vaccine (DTP3); among them, 9.6 million (44 %) started, but did not complete, DPT 3-dose series [2, 3].

Despite the fact that Africa has made remarkable progress in immunization services, large numbers of children remain unvaccinated and under-vaccinated. The performance of routine immunizations in the African Region has stalled during the last decade for the majority of vaccine delivered-antigens. According to a 2013 immunization data report, vaccine coverage was 75 %; and Ethiopia has the second largest number of incompletely vaccinated children from the region, next to Nigeria [3–5].

Ethiopia’s Federal Ministry of Health (FMOH) adopted WHO’s recommended vaccination schedule for developing countries. A child is considered fully vaccinated if he/she has received a Bacillus Callmete Guerin (BCG) vaccination against tuberculosis; three doses of pentavalent vaccine (DPT-Hep B-Hi-b) to prevent diphtheria, pertussis, tetanus, *Haemophilus influenzae* type b and hepatitis B; at least three doses of polio vaccine; and one dose of measles vaccine. Recently, a new 10-valent pneumococcal conjugate vaccine (PCV) and a rotavirus vaccine were introduced into the routine infant immunization schedule and they are administered with the existing pentavalent vaccine (Table 1). It is also recommended that children receive the complete schedule of vaccinations before their first birthday and that the vaccinations be recorded on a vaccination card that is given to the parents or guardians [6–8].

**Table 1:** Routine immunization schedule in Ethiopia, 2014

Vaccine	Disease	Age
BCG	Tuberculosis	At Birth
Pentavalent	Diphtheria, Pertussis, Tetanus, H. influenza type b, Hepatitis B	6, 10, 14 weeks
OPV	Polio	At Birth, 6, 10, 14 weeks
Measles	Measles	9 Months
Pneumonia-conjugate Vaccine (PCV)	Pneumonia	6, 10, 14 weeks
Rotarix (rotavirus vaccine)	Rotavirus	6, 10 weeks
Tetanus (TT) immunization for women in child bearing age	Tetanus	1st contact pregnancy; +1 month, +6 months; +1 year, +1 year

Based on the Ethiopian Demographic and Health Survey (EDHS) 2011 report, 15 % of children in Ethiopia haven’t received any vaccinations, and 56 % were vaccinated againest measles. The coverage level of pentavalent 3 was 44.3 %, as assessed by card and history. This is a significant improvement compared to EDHS 2005 performances. However, the full immunization rate is only 24 % and pentavalent 3 coverage in many regions of the country is below 80 % [9]. Multiple studies demonstrate that the EPI schedule in Ethiopia is not completed as planned [10–14].

To improve the rate of full immunization coverage, we have to investigate and overcome the reasons for incomplete immunization status of children. Factors of incomplete childhood immunization are poorly understood and few Ethiopian community-based studies are available. Most of the studies were cross-sectional surveys and assessed only EPI coverage. They also focused on individual and community factors with less consideration of factors related to service delivery. The objective of this study was to identify both individual and service performance related factors associated with incomplete childhood immunization in the Arbegona district, southern Ethiopia.

## Methods

### Study area

**Fig. 1:**
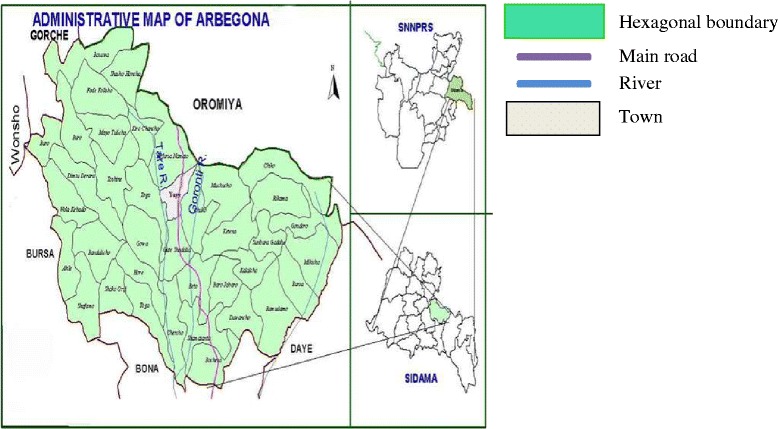
Map of Arbegona district, Sidama zone, SNNPR, Ethiopia. Copyright (Arbegona District Administrative Office)

The community-based, unmatched case-control study was conducted from January 15 to February 15, 2014 in the Arbegona district, which is one of the 19 districts in Sidama zone, SNNPRS (Southern Nations, Nationalities and Peoples Regional State), Ethiopia. The district is located 74 km and 349 km from Hawassa (capital city of SNNPRS) and Addis Ababa, respectively and is divided into 1 urban and 38 rural *kebeles* (lower administrative units) (Figure 1). The population is estimated to be 166,017.

### Study participants

Children aged 12 to 23 months with a history of vaccination were eligible for the study. The cases were children aged 12 to 23 months who did not complete the recommended schedule of immunization and the controls were any eligible child who had completed the recommended schedule of immunization.

The sample size was generated using Epi-Info 6.04 statistical software (CDC, Atlanta, 2005) and calculated using the mother’s educational level as the key indicator. This choice is supported by a previous study observing a significant association between a mother’s educational level and the immunization status of the child [15]. Setting the case to control ratio of 1:2, a design effect of 2, and a non-response rate of 10 % resulted in the total sample size required for the study to be 548 (cases =183 and controls = 365).

Multistage sampling was used to obtain a representative study sample. There are a total of 38 rural kebeles and 1 urban kebele in the district. In the first stage, simple random sampling was used to select 14 kebeles; the urban kebele was also included. In each selected kebele, a list of cases and controls and their full addresses were pulled from the records of nearby health centers and health posts that provide different child health services, including EPI services. From this sampling frame, a final subset of cases and controls was selected from each kebele based on a probabilty proportional to the number of the children under one year of age in each kebele and mothers of the selected children were traced to participate in the study (Additional file 1).

### Data collection procedure

The data were collected using a structured questionnaire adapted from a previous study [14]. Before undertaking data collection, the questionnaire was pretested on other similar population taking 5 % of the total sample and necessary modifications were made. The questionnaire was prepared originally in English and translated to the local language (Sidamgna). The questionaire was further translated from Sidamgna to English to check for consistency. The primary respondent was the mother of the child. In case of her absence, the questionnaire could also be completed by the father or another adult in the household acting as the primary caregiver (Additional file 2). The data were collected by trained, diploma nurses, fluent in Sidamgna, using the structured questionnaire translated to Sidamgna. Three health professionals with bachelors degree in health supervised the data collection process. Before data collection, the data collectors and supervisors were trained thoroughly.

Factors affecting immunization service delivery were qualitatively determined based on feedbacks from Focus Group Discussions (FGDs) and in-depth interviews. A total of five FGDs were independently conducted with two FGDs consisting of groups of nine health professionals and three FGDs consisting of ten randomly selected community health workers (Health Extension Workers). In-depth interviews were also conducted with the head of the District Health Office, heads of Health Centers and District Health Office experts, selected due to their direct or indirect role in implementing the EPI program in the district. A semi-structured, open-ended guide (Additional file 3) and in-depth interview guide (Additional file 4) were used in FGDs and in-depth interviews, respectively.

### Data processing and analysis

Data were entered and analyzed using SPSS-version 16 statistical software. Descriptive statistics were used to summarize the data, and a bivariate analysis was carried out to describe associations between exposure variables and childhood immunization status and assessed by chi-square and odds ratio with a significance level of *p*-value ≤ 0.05. Variables with *p*-value ≤ 0.25 were included in the final model. In the multivariable analysis, backward stepwise logistic regression with *p*-value ≤ 0.05 was applied to identify independent predictors of incomplete childhood immunization. The p-values were based on the likelihood ratio test which is the recommended test statistic in using backward stepwise logistic regression.

The qualitative data were transcribed and analyzed using thematic framework and the results were triangulated with the quantitative findings to assess key factors in incomplete vaccination status.

### Operational definitions

- ▪ *Attitude towards the service delivered:* This was measured using four items where an attitude score > 10 labelled is as ‘positive’ and a score ≤ 10 is labelled as ‘negative’.
- ▪ *BCG to Measles dropout rate:* The percent of children vaccinated for BCG who did not receive measles vaccines.
- ▪ *Complete (full) immunization:* The childhood immunization status once a child has recieved all recommended vaccines, including BCG, three doses of pentavalent, three doses of polio and measles vaccines by the age of 12 months.
- ▪ *Dropout rate (DOR):* The percent of children who received the initial vaccine (BCG or pentavalent 1) but not the final vaccines (pentavalent 3 or measles).
- ▪ *Incomplete (partial) immunization/Defaulter:* The childhood immunization status if the child missed at least one of the recommended vaccines.
- ▪ *Knowledge about immunization:* If the mother mentioned at least one type of vaccination and was aware of at least one EPI targeted disease that can be prevented by immunization.
- ▪ *Knowledge about a schedule of immunization:* If the mother knew the schedule for at least one type of vaccine.
- ▪ *Missed opportunities:* If an eligible child visited the selected health facility or outreach site but didn’t receive any or all of the vaccines for which he/she is eligible.
- ▪ *Pentavalent 1 to pentavalent 3 dropout rate:* The percent of children vaccinated for pentavalent 1 who did not receive pentavalent 3.
- ▪ *Perception concerning vaccine side effects:* This was measured using the Likert scale with ‘agree’ and ‘strongly agree’ responses categorized as ‘positive perception’ and ‘disagree’ and ‘strongly disagree’ categorized as ‘negative perception’.

### Ethical consideration

Ethical clearance was obtained from Health Research and Postgraduate Studies, coordinating office of Jimma University and was communicated to the Regional Health Bureau and to the District Health Office. Prior to data collection, the purpose of the study was clearly explained and written consent was obtained from the mothers. Qualitative data was collected after securing informed verbal consent from every participant.

## Results

From the total selected 183 cases and 365 controls, 182 of the cases and 362 of the controls participated in the study with a response rate of 99.45 % and 99.17 % among cases and controls, respectively. For 154 (84.6 %) of the cases and 294 (81.2 %) of the controls, mothers were primary respondents. For the rest, 28 (15.3 %) of the cases and 68 (18.7 %) of the controls, the father or another adult in the household acting as a primary caregiver, acted as respondents. The measles vaccine was the most defaulted vaccine, with a vaccination rate of only 10.9 % among cases. The BCG vaccination rate was 90 % among cases and the pentavalent and BCG to measles dropout rates were 51.13 % and 87.7 %, respectively.

### Socio-demographic variables

From the total 182 children who didn’t complete their immunization (cases), males account for 107 (58.8 %) and females account for 75 (41.2 %). The percent of males in the control group was 76.7 %. The mean age of the cases was 19.5 months (Standard Deviation (SD) = 3.6) and 18.3 months (SD = 3.4) for controls. Most mothers, of both case and control children, were Sidama by ethnicity, Protestant by religion and married. Almost half of the mothers (49 %) of both groups were farmers. The median age of the mothers/caretakers was 27 years (SD = 6.9) and 28 years (SD = 6.0), for cases and controls respectively. Mothers of case children had a median monthly family income of 400 Ethiopian Birr (ETB) or 19.56 United States Dollar (USD) while mothers of control children had a median monthly family income of 450 ETB or 22 USD. In the bivariate analysis, maternal age, family size and birth order were significant at p-value < 0.05 from the different social-demographic variables (Table 2).

**Table 2:** Results from bivariate analysis of socio-demographic factors related to incomplete childhood immunization in Arbegona district, southern Ethiopia, 2014

Variables	Category	Cases	Controls	Crude OR	*P*-value
		No (%)	No (%)	(95 % CI)	
Maternal	≤19	81 (44.5)	32 (8.9)	8.27 (5.19, 13.18)	<0.001*
Age (In years)	>19	101 (55.5)	330 (91.1)	1	
Occupational status	Housewife	56 (30.7)	118 (32.9)	0.92 (0.61, 1.39)	0.71
	Farmer	90 (49.4)	176 (49.1)	1	
	Merchant	14 (7.7)	34 (9.5)	0.80 (0.41, 1.57)	0.52
	Other	22 (12)	30 (8.3)	1.43 (0.78, 2.62)	0.24
Monthly income (In ETB)	≤500	122 (67)	217 (59.9)	1	
	500 – 1000	38 (20.8)	101 (27.9)	0.67 (0.43, 1.03)	0.07
	≥1000	22 (12)	44 (12.1)	0.89 (0.50, 1.55)	0.68
Mother’s educational status	Illiterate	116 (63.7)	226 (62.4)	1	
	Read and write	13 (7.1)	23 (6.3)	1.10 (0.53, 2.25)	0.79
	Elementary	39 (21.4)	75 (20.7)	1.01 (0.64, 1.58)	0.95
	Secondary and above	14 (7.7)	38 (10.5)	0.71 (0.37, 1.37)	0.31
Father’s educational status	Illiterate	60 (33.3)	133 (36.7)	1	
	Read and write	29 (16.1)	53 (14.6)	1.21 (0.70, 2.09)	0.48
	Elementary	52 (28.8)	98 (27)	1.17 (0.74, 1.85)	0.48
	Secondary and above	39 (21.6)	78 (21.5)	1.10 (0.67, 1.81)	0.68
Residence	Rural	160 (87.9)	320 (88.4)	1	
	Urban	22 (12.1)	42 (11.6)	1.04 (0.60, 1.81)	0.86
Family size	≤5	54 (29.6)	175 (48.3)	0.45 (0.30, 0.65)	<0.001*
	>5	128 (70.4)	187 (51.7)	1	
Child sex	Male	107 (58.8)	201 (55.5)	1	
	Female	75 (41.2)	161 (44.5)	0.87 (0.60, 1.24)	0.46
Birth order	1	20 (11.1)	119 (32.8)	1	
	2 – 4	92 (51.1)	153 (42.2)	3.57 (2.08, 6.13)	<0.001*
	≥5	68 (37.7)	90 (24.8)	4.49 (2.54, 7.94)	<0.001*
Birth interval (In Months)	≤23	51 (31.4)	66 (27.3)	1.18 (0.75, 1.86)	0.4
	24 – 47	93 (57.4)	143 (59)	1	
	>47	18 (11.2)	33 (13.6)	0.83 (0.44, 0.57)	0.5

*Statistically significant at *p* < 0.05

### Mother’s knowledge and attitude about immunization

Of the mothers, 101 (55.5 %) mothers of the cases and 263 (72.6 %) of the controls knew about immunization; only half of the mothers of both groups knew the schedule of at least one type of vaccine.

The proportion of mothers who knew the schedule of BCG vaccine was almost the same between cases (33 %) and controls (34 %). However, 81 (30 %) and 23 (22 %) mothers of the cases and controls who knew about immunization, respectively, knew the measles vaccine. Almost all mothers of the cases (98.9 %) and control (98.2 %) didn’t know the polio or pentavalent vaccine schedules. From those mothers of the cases who knew about immunization, 38 % of them reported that they knew whether or not their child completed the immunization schedule by following health professional’s/Health Extension Worker’s (HEW’s) instructions. Half of the mothers from both groups had a positive attitude towards the last received child immunization service. Mothers’ knowledge about immunization and immunization benefits were significantly associated with completion of child immunization schedule in the bivariate analysis (Table 3).

**Table 3:** Results from bivariate analysis of mother’s knowledge and attitude about immunization in Arbegona district, southern Ethiopia, 2014

Variables	Category	Cases	Controls	Crude OR	*P*-value
		No (%)	No (%)	(95 % CI)	
Knew about immunization	Yes	101 (55.5)	263 (72.6)	1	
	No	81 (44.5)	99 (27.3)	2.13 (1.46, 3.09)	<0.001*
Knew the schedule of vaccines	Yes	50 (49.5)	134 (51)	1	
	No	51 (50.5)	129 (49)	1.06 (0.67, 1.67)	0.8
Knew the benefits of immunization	Yes	88 (87.9)	256 (97.7)	1	
	No	13 (12.1)	6 (2.3)	6.30 (2.32, 17.08)	<0.001*
Knew about vaccine side effects	Yes	25 (24.8)	47 (18)	1.49 (0.85, 2.56)	0.16
	No	76 (75.2)	214 (82)	1	
Attitude towards benefits of immunization	Positive	165 (94.8)	339 (96.6)	1	
	Negative	9 (5.2)	12 (3.4)	1.54 (0.63, 3.73)	0.33
Perception about vaccine side effects	Positive	104 (60.1)	236 (65.9)	1	
	Negative	69 (39.8)	122 (34.1)	1.28 (0.86, 1.88)	0.21
Attitude towards the last received child immunization	Positive	90 (49.7)	176 (49)	1	
	Negative	91 (50.3)	183 (51)	0.97 (0.68, 1.39)	0.8

*Statistically significant at *p* < 0.05

### Factors related to immunization service delivery

According to the mothers’ report, 74 (40.6 %) mothers of the cases and 130 (35.6 %) of controls took their child to a health institution for child health services other than immunization. Of those, 63 (85 %) mothers of the cases and 116 (89 %) of the controls were advised to vaccinate their child. Thus, the missed opportunity rate was 14.9 % and 10.8 % among cases and controls, respectively (Table 4).

**Table 4:** Results from bivariate analysis of factors related to immunization service delivery in Arbegona district, southern Ethiopia, 2014

Variables	Category	Cases	Controls	Crude OR	*P*-value
		No (%)	No (%)	(95 % CI)	
Advised to vaccinate their child during their health institution visit	Yes	63 (85.1)	116 (89.2)	1	
	No	11 (14.9)	14 (10.8)	1.44 (0.62, 3.37)	0.39
Information was received about the next vaccine schedule	Yes	172 (95.6)	352 (97.5)	1	
	No	8 (4.4)	9 (2.5)	1.81 (0.69, 4.79)	0.22
Availability of immunization clinic	Yes	145 (79.7)	287 (79.3)	1	
	No	37 (20.3)	75 (20.7)	0.97 (0.62, 1.51)	0.91
Difficulty of getting immunization shots	Yes	39 (21.7)	75 (20.7)	1.05 (0.68, 1.63)	0.79
	No	141 (78.3)	287 (79.3)	1	
Convenient hours for immunization	Yes	63 (34.6)	224 (62.9)	1	
	No	119 (65.4)	132 (37.1)	0.70 (0.48, 1.04)	0.08
Postponing of the immunization schedule	Yes	63 (34.6)	79 (21.8)	1.89 (1.27, 2.81)	0.001*
	No	119 (65.4)	283 (78.2)	1	

*Statistically significant at *p* < 0.05

Only 9 (6 %) mothers of the cases and 26 (9 %) of the controls gave birth at health institutions, and all mothers of both groups were advised to vaccinate their child after delivery. Almost all mothers of the cases (97.4 %) and controls (97 %) who attended postnatal care (PNC) were also advised to vaccinate their child during their PNC visit. Based on the report of mothers who postponed their child’s immunization, unavailability of the vaccine and absence of vaccinators were reasons for postponing the immunization schedule.

Different service delivery related factors, including missed opportunity, were examined and postponing of the immunization schedule was the only significant variable in the bivariate analysis (Table 4).

### Independent predictors of incomplete childhood immunization

Knowledge about immunization was removed in the multivariable analysis from further analysis due to its co-linearity with other variables. Maternal age, birth order and knowledge about the benefits of vaccination retained their statistical significance after adjusting for other variables. There is a marginal significant association (*p*-value = 0.05) between a mother’s perception about vaccine side effects and immunization status of children after adjusting for confounders. The risk of defaulting their child’s vaccine series is higher in younger mothers than older mothers (AOR = 9.54; 95 % CI = 5.03, 18.09). Child birth order was found to be associated with immunization incompletion; being second to fourth in the family (AOR = 3.64; 95 % CI = 1.63, 8.14) and being fifth and above in the family (AOR = 5.27; 95 % CI = 2.20, 12.64) had a higher likelihood to default than being born first. Mothers who didn’t know the benefits of vaccination were five times more likely to have defaulter children than their counterparts (AOR = 5.51; 95 % CI = 1.52, 19.94). The risk of not completing child immunization also increases in mothers with negative perceptions of vaccine side effects (AOR = 1.92; 95 % CI = 1.01, 3.70) (Table 5).

**Table 5:** Determinants of incomplete childhood immunization status in Arbegona district, southern Ethiopia, 2014

Variables	Category	Adjusted OR (95 % CI)	*P*-value
Maternal Age (In years)	≤19	9.54 (5.03, 18.09)	<0.001*
	>19	1	
Monthly income (In ETB)	≤500	1	
	500 – 1000	0.49 (0.23, 1.05)	0.07
	>1000	1.59 (0.65, 3.85)	0.30
Family size	≤5	0.70 (0.34, 1.45)	0.34
	>5	1	
Birth order	1	1	
	2 – 4	3.64 (1.63, 8.14)	<0.001*
	≥5	5.27 (2.20, 12.64)	0.002*
Knew the benefits of immunization	Yes	1	
	No	5.51 (1.52, 19.94)	0.009*
Knew about vaccine side effects	Yes	0.96 (0.46, 2.01)	0.90
	No	1	
Perception about vaccine side effects	Positive	1	
	Negative	1.92 (1.01, 3.70)	0.05*
Information was received about the next vaccine schedule	Yes	1	
	No	0.20 (0.01, 2.45)	0.21
Convenient hours for immunization	Yes	1	
	No	1.06 (0.55, 2.04)	0.80
Postponing of the immunization schedule	Yes	1.30 (0.70, 2.41)	0.39
	No	1	

*Statistically significant at *p* < 0.05

### Evidence besides figures

Focus group discussions were held with HEWs and health professionals independently. During the discussion with HEWs, the participants indicated that mothers fear some common vaccine side effects, even if they are advised of vaccine side effects. As a result, they may postpone, or not come back for, the next scheduled vaccination when they see common vaccine reactions. They also noted that last year, vaccine stock-outs were encountered on multiple occasions. One HEW stated that *“there were occasions in which we mobilize the community for immunization campaign and vaccines were not available when mothers came to immunize their child, and this is a big challenge and mothers may refrain from vaccinating their child in other immunization campaigns”.* The discussants also stated that some mothers migrate from one place to another within the district and this favours a default on their child’s immunization. The participants stressed that when this occurs, it is difficult to trace these mothers and they may not complete their child’s immunization in their new residence.

In the discussion with health professionals, they agreed that most health professionals are aware of the internal referral system. They check the immunization status of children who come for other child health services and refer to the immunization room when necessary. Most health professionals also advise mothers who come for maternity services to vaccinate their child. Most of the participants, however, said that there are gaps from professionals in informing parents about common vaccine side effects. They also mentioned that mothers’ knowledge of, and compliance with, the immunization schedule is very poor.

In-depth interviews were conducted with the Head of District Health Office, District Health Office experts and Heads of Health Centers. The interviewees reported that vaccine stock-outs and mothers’ relocating are the main problems in the EPI service delivery and are possible reasons for incomplete immunization status of children. Heads of the Health Centers also felt that there are gaps in health professionals informing mothers of vaccine side effects and the schedule of subsequent vaccines.

Migration of mothers and unavailability of vaccines on the appointed immunization dates were found as major reasons for partial immunization of children by the qualitative method.

## Discussion

This study was done in the Arbegona district, southern Ethiopia, to identify factors associated with incomplete childhood immunization. The study assessed different factors and found that a child’s birth order, as well as the mother’s age, knowledge about immunization benefits and perception of vaccine side effects, were determinant factors of incomplete child immunization.

The BCG vaccination rate (90 %) among children who didn’t complete the full vaccine schedule was higher in the study area as compared to the study conducted in Wonago district, south Ethiopia, which was 73.5 % among cases [14]. In our study, however, the measles vaccination rate was only 10 % among cases, lower than the vaccination rate found in Wonago district where the rate was 17 % among cases. The BCG to measles dropout rate among cases was also higher than was found in Wonago district, which was 76.2 % among cases [14]. This was due to the high BCG and low measles vaccination rate among cases in the Arbegona study area.

Younger mothers were at a high risk of defaulting on their child’s immunizations than older mothers. It is well recognized that age plays an important role in womens’ utilization of health services and maternal age may sometimes serve as a proxy for accumulated knowledge of health care services. Age may be a factor, which may have a positive influence on accepting the full immunization of children. This may be due to older mothers having more knowledge about health care services and valuing the full immunization of their children more than younger mothers [10, 16, 17].

The birth order of the child was the other determinant factor of incomplete immunization status. Being second and later born in the family showed a strong association with vaccination incompletion. As the number of children in the family increases, family resources, including time and attention, are shared among the children. This may result in children born late in the family not getting the full vaccine series. This finding is in line with similar previous studies [15, 18].

Mothers who didn’t know the benefits of immunization were five times more likely to have defaulter children than mothers who knew the benefits of immunization. Other studies have also shown that a mother’s awareness of the importance of vaccination has a strong association with complete immunization status of children [13, 14]. Other researchers have found that a mother’s knowledge of the immunization schedule has a strong association with complete immunization status of children [11, 12]. In this study, however, a mother’s knowledge of immunization schedule was not a predictor of incomplete immunization status. Mothers knew whether their child completed the immunization schedule or not by following health professional’s/HEW’s instructions and may follow the schedule without knowing which vaccine the schedule is for. Mothers who don’t know the benefits of immunization may be reluctant in following health professional’s/ HEW’s instructions concerning vaccine schedules, whether they know the schedule of each vaccine or not.

Our study also found that children of mothers with a fear of common vaccine side effects were at a higher risk of defaulting than children of mothers who perceived common vaccine side effects positively. A qualitative study on the behavioural factors of immunization in Uganda reported that mothers who are afraid of vaccine side effects either decline or delay subsequent immunizations [19]. Although modern vaccines are safe, no vaccine is entirely without risk. When some children experience mild side effects, their mothers may refuse further immunizations for their children [20, 21].

From the qualitative study, the migration of mothers from one place to another was the main reason for inadequate immunization of children. A study done in southern Ethiopia reported that children born to rural–rural migrant women are less likely to get immunized compared to children born to non-migrant women residing in rural areas [22]. Migration might be associated with a high risk of defaulting due to the weak social integration and low vaccine uptake of migrating populations. It is also difficult for the health sector to trace these children and enable their mothers to complete their child immunization [23, 24].

Unavailability of vaccines on the appointed dates due to vaccine stock-outs, was another reason of incomplete immunization status found by the qualitative study. Vaccine unavailability was also described by most of the mothers for postponing the immunization schedule. A study done in the Sinana district, southeast Ethiopia, also reported that 52.1 % of mothers returned home without vaccinating their child due to a lack of vaccines in health facilities [18]. When mothers came to immunize their child and were denied the service because of a vaccine shortage, they are unlikely to bring their child back for vaccination [21, 25].

## Conclusions

Children of younger mothers, children born late in the family, children of mothers who don’t know the benefits of immunization, and children of mothers with negative perceptions of vaccine side effects were significantly associated with an incomplete immunization status. The qualitative approach revealed that the migration of mothers and vaccine stock-outs were key reasons related to partial immunization of children.

EPI performance problems in African countries are multi-faceted and primarily related to health system issues. In order to address the identified problems, understanding the local context of immunization programs is of great importance for identifying effective and evidence-based interventions [4, 26]. To reduce the number of children with an incomplete immunization status, the district needs to consider specific planning for mothers with risk profiles, and focus on strengthening health communication activities to increase immunization awareness and address concerns of vaccine side effects at community level. This can be accomplished through integrating the EPI service to other elements of primary health care.

## Additional files

Additional file 1: **STROBE Statement.** (PDF 176 kb)

Additional file 2: **Structured questionnaire for mothers/caregivers.** (PDF 156 kb)

Additional file 3: **Focus Group Discussion guide.** (PDF 78 kb)

Additional file 4: **In-depth interview guide.** (PDF 79 kb)

## Abbreviations

AOR: adjusted odds ratio
BCG: Bacillus Callmete Guerin
CDC: centers for disease control
CI: confidence interval
COR: crude odds ratio
EDHS: Ethiopian Demographic and Health Survey
EPI: Expanded Program on Immunization
ETB: Ethiopian Birr
FGD: focus group discussion
HEW: Health Extension Worker
PNC: postnatal care
SNNPRS: Southern Nations, Nationalities and Peoples Regional State
WHO: World Health Organization
