# Supplementary material for: Factors associated with incomplete childhood immunization in Arbegona district, southern Ethiopia: a case – control study
Source: BMC Public Health. 2016 Jan 12;16:27. doi: 10.1186/s12889-015-2678-1 (PMC4711011; doi:10.1186/s12889-015-2678-1)
Supplement: Supplementary file 1 — STROBE Statement. (PDF 176 kb) [file 12889_2015_2678_MOESM1_ESM.pdf]

STROBE Statement—Checklist of items that should be included in reports of ***case-control studies***

	Item No	Recommendation
**Title and abstract**	1	**Title:** Factors associated with incomplete childhood immunization in Arbegona district, southern Ethiopia: A case – control study
		**Abstract:**<br><br>**Background:** The prevention of child mortality through immunization is one of the most cost-effective and widely applied public health interventions. In Ethiopia, the Expanded Program on Immunization (EPI) schedule is rarely completed as planned and the full immunization rate is only 24%. The objective of this study was to identify determinant factors of incomplete childhood immunization in Arbegona district, Sidama zone, southern Ethiopia.<br><br>**Methods:** A community based unmatched case-control study was undertaken among randomly selected children aged 12 to 23 months and with a total sample size of 548 (183 cases and 365 controls). A multi-stage sampling technique was used to get representative cases and controls. Data was collected using a structured questionnaire and analyzed using SPSS version 16 statistical software. Bivariate and multiple logistic regression analyses were done to identify independent factors for incomplete immunization status of children. Qualitative data were also generated and analyzed using thematic framework.<br><br>**Results:** The incomplete immunization status of children was significantly associated with young mothers (AOR = 9.54; 95% CI = 5.03, 18.09), being born second to fourth (AOR = 3.64; 95% CI = 1.63, 8.14) and being born fifth or later in the family (AOR = 5.27; 95% CI = 2.20, 12.64) as compared to being born first, a mother's lack of knowledge about immunization benefits (AOR = 5.51; 95% CI = 1.52, 19.94) and a mother's negative perception of vaccine side effects (AOR = 1.92; 95% CI = 1.01, 3.70). The qualitative finding revealed that the migration of mothers and unavailability of vaccines on appointed immunization dates were the major reasons for partial immunization of children.<br><br>**Conclusion:** To reduce the number of children with incomplete immunization status, the Arbegona district needs to consider specific planning for mothers with these risk profiles. A focus on strengthening health communication activities to raise immunization awareness and address concerns of vaccine side effects at community level is also needed. This could be achieved through integrating the immunization service to other elements of primary health care.
**Introduction**		
Background/ rationale	2	World-wide, immunization programs have had a tremendous impact on the prevalence of many life-threatening diseases. However, inadequate levels of immunization against childhood diseases remain a significant public health problem in resource-poor areas of the globe (page 3).<br>Ethiopia is one of the countries with large number of incompletely vaccinated children and Penta 3 coverage in many regions of the country is below 80% (page 3 – 4).<br>The effectiveness of immunization programs in resource-poor settings can be influenced by factors related to the vaccines in use, to health services or to communities. The relative effect of

		each one of the above factors may significantly vary according to geographical areas. In this context, the understanding of local hurdles for effective immunization programs is crucial to develop and implement appropriate solutions (page 4).
Objectives	3	- To examine socio-demographic characteristics among children with complete and incomplete immunization status in the Arbegona district, southern Ethiopia.<br>- To examine mother's knowledge and attitude about immunization among children with complete and incomplete immunization status in the Arbegona district, southern Ethiopia.<br>- To examine factors related to immunization service delivery among children with complete and incomplete immunization status in the Arbegona district, southern Ethiopia.<br>- To identify factors associated with incomplete childhood immunization in the Arbegona district, southern Ethiopia.
**Methods**		
Study design	4	Community-based, unmatched case-control study (page 4)
Setting	5	**Setting:** The study was conducted in Arbegona district which is one of the 19 districts in Sidama zone, SNNPRS, Ethiopia. (page 4)<br>**Period of recruitment:** September, 2013 – June, 2014<br>**Period of data collection:** January, 2014 – February, 2014
Participants	6	**Eligibility criteria:** – Child age – 12 to 23 months<br>– History of vaccination<br>**Cases:** Eligible Child who did not complete the recommended schedule of immunization.<br>**Controls:** Eligible child who had completed the recommended schedule of immunization.<br>**Sources of case and control selection:** A list of cases and controls were generated from Health facility records (EPI registration books). The required number of cases and controls was selected based on a probabilty proportional to the number of the children under one year of age in each selected *kebele*. (page 5)
Variables	7	**Outcome variable:** Childhood immunization status<br>**Exposure variables:**<br>- ***Socio-demographic factors:*** Mother's educational status, Mother's age, Family income, Sex of the child, Family size, Birth order, Birth interval, Father's educational status, Occupational status, Place of residence (rural/urban).<br>- ***Mother's knowledge and attitude about immunization:*** Knowledge about benefits of immunization, Knowledge about EPI targeted diseases, Knowledge about vaccine side effects, Knowledge about schedule of vaccines, Attitude towards benefits of immunization, Perception about vaccine side effects, Attitude towards the service delivery<br>- ***Factors related to immunization service delivery:*** Missed opportunity, Information delivery to mothers about immunization completion, Vaccine shortage/unavailability, Availability of immunization clinic, Integration of the immunization service with other services, Convenience of the immunization service, Postponing of the immunization schedule

Data sources/ measurement	8*	For both cases and controls, quantitative data were collected using a pre-tested structured questionnaire. Qualitative methods (Focus Group Discussions and In-depth interviews) were employed to assess some factors related to immunization service delivery, which couldn't be assessed quantitatively. (page 5 – 6)
Bias	9	The data were collected by trained diploma nurses who have experience of collecting a research data and the data collection process was closely monitored for data quality through regular field visits. (page 6)<br>The data collectors had no information about the immunization status of the children.
Study size	10	The sample size was generated using Epi-Info 6.04 statistical software (CDC, Atlanta, 2005) using the mother's educational level, which gives maximum sample size as compared to other variables to bring difference in the two groups.<br>The following parameters were used to calculate the sample size:<br>Confidence interval (CI): 95%<br>Power of test : 80%<br>Case to Control ratio: 1: 2<br>Non-response rate: 10%<br>Design effect: 2<br>The total number of the study participants was 548.<br>Total number of cases – 183<br>Total number of controls – 365 (page 5)
Quantitative variables	11	Standard groupings, used in other similar published studies, were considered in the analyses. Operational definitions of some variables were also used and the categories were defined by considering the context of the study area (literacy status of the mothers, the difficulty in getting health information, EPI coverage of the district etc….). (page 7)
Statistical methods	12	Data were coded, entered, cleaned, explored and finally analyzed using SPSS-version 16 statistical software.<br>First descriptive statistics were used to summarize the data, and a bivariate analysis was carried out to see the presence and degree of statistical association between the outcome and explanatory variables. In the multivariable analysis, backward stepwise logistic regression with p-value $\leq$ 0.05 was applied to identify independent predictors of incomplete childhood immunization, through controlling of potential confounding factors. (page 6)
**Results**		
Participants	13*	**Cases:**<br>Confirmed eligible and included in the study: 183<br>Participated in the study and analysed: 182 (1 mother was not volunteer to participate)<br>**Controls:**<br>Confirmed eligible and included in the study: 365<br>Participated in the study and analysed: 362 (3 mothers were not volunteer to participate)

Descriptive data	14*	**Cases:** (Table 2, page 22)

Variables	Category	No (%)	Missing data
Maternal Age	≤ 19	81 (44.5)	
(In years)	>19	101 (55.5)	
Occupational status	Housewife	56 (30.7)	
	Farmer	90 (49.4)	
	Merchant	14 (7.7)	
	Other	22 (12)	
Monthly income	≤ 500	122 (67)	
(In Ethiopian Birr)	500 - 1000	38 (20.8)	
	≥ 1000	22 (12)	
Mother's educational status	Illiterate	116 (63.7)	
	Read and write	13 (7.1)	
	Elementary	39 (21.4)	
	Secondary and above	14 (7.7)	
Father's educational status	Illiterate	60 (33.3)	2
	Read and write	29 (16.1)	
	Elementary	52 (28.8)	
	Secondary and above	39 (21.6)	
Residence	Rural	160 (87.9)	
	Urban	22 (12.1)	
Family size	≤ 5	54 (29.6)	
	> 5	128 (70.4)	
Child sex	Male	107 (58.8)	
	Female	75 (41.2)	
Birth order	1	20 (11.1)	2
	2 - 4	92 (51.1)	
	≥ 5	68 (37.7)	
Birth interval	≤ 23	51 (31.4)	
(In Months)	24 – 47	93 (57.4)	
	> 47	18 (11.2)	

Controls: (Table 2, page 22)

Variables	Category	No (%)	Missing data
Maternal Age (In years)	≤ 19	32 (8.9)	
	>19	330 (91.1)	
Occupational status	Housewife	118 (32.9)	4
	Farmer	176 (49.1)	
	Merchant	34 (9.5)	
	Other	30 (8.3)	
Monthly income (In ETB)	≤ 500	217 (59.9)	
	500 - 1000	101 (27.9)	
	≥ 1000	44 (12.1)	
Mother's educational status	Illiterate	226 (62.4)	
	Read and write	23 (6.3)	
	Elementary	75 (20.7)	
	Secondary and above	38 (10.5)	
Father's educational status	Illiterate	133 (36.7)	
	Read and write	53 (14.6)	
	Elementary	98 (27)	
	Secondary and above	78 (21.5)	
Residence	Rural	320 (88.4)	
	Urban	42 (11.6)	
Family size	≤ 5	175 (48.3)	
	> 5	187 (51.7)	
Child sex	Male	201 (55.5)	
	Female	161 (44.5)	
Birth order	1	119 (32.8)	
	2 - 4	153 (42.2)	
	≥ 5	90 (24.8)	
Birth interval (In Months)	≤ 23	66 (27.3)	
	24 – 47	143 (59)	
	> 47	33 (13.6)	

Outcome data	15*	**Cases:**

Mother's knowledge and attitude about immunization (Table 3, page 24)

Variables	Category	No (%)
Knew about immunization	Yes	101 (55.5)
	No	81 (44.5)
Knew the schedule of vaccines	Yes	50 (49.5)
	No	51 (50.5)
Knew the benefits of immunization	Yes	88 (87.9)
	No	13 (12.1)
Knew about vaccine side effects	Yes	25 (24.8)
	No	76 (75.2)
Attitude towards benefits of immunization	Positive	165 (94.8)
	Negative	9 (5.2)
Perception about vaccine side effects	Positive	104 (60.1)
	Negative	69 (39.8)

Factors related to immunization service delivery (Table 4, page 25)

Variables	Category	No (%)
Advised to vaccinate their child during their health institution visit	Yes	63 (85.1)
	No	11 (14.9)
Information was received about the next vaccine schedule	Yes	172 (95.6)
	No	8 (4.4)
Availability of immunization clinic	Yes	145 (79.7)
	No	37 (20.3)
Difficulty of getting immunization shots	Yes	39 (21.7)
	No	141 (78.3)
Convenient hours for immunization	Yes	63 (34.6)
	No	119 (65.4)
Postponing of the immunization schedule	Yes	63 (34.6)
	No	119 (65.4)

Controls:

Mother's knowledge and attitude about immunization (Table 3, page 24)

Variables	Category	No (%)
Knew about immunization	Yes<br>No	263 (72.6)<br>99 (27.3)
Knew the schedule of vaccines	Yes<br>No	134 (51)<br>129 (49)
Knew the benefits of immunization	Yes<br>No	256 (97.7)<br>6 (2.3)
Knew about vaccine side effects	Yes<br>No	47 (18)<br>214 (82)
Attitude towards benefits of immunization	Positive<br>Negative	339 (96.6)<br>12 (3.4)
Perception about vaccine side effects	Positive<br>Negative	236 (65.9)<br>122 (34.1)

Factors related to immunization service delivery (Table 4, page 25)

Variables	Category	No (%)
Advised to vaccinate their child during their health institution visit	Yes<br>No	116 (89.2)<br>14 (10.8)
Information was received about the next vaccine schedule	Yes<br>No	352 (97.5)<br>9 (2.5)
Availability of immunization clinic	Yes<br>No	287 (79.3)<br>75 (20.7)
Difficulty of getting immunization shots	Yes<br>No	75 (20.7)<br>287 (79.3)
Convenient hours for immunization	Yes<br>No	224 (62.9)<br>132 (37.1)
Postponing of the immunization schedule	Yes<br>No	79 (21.8)<br>283 (78.2)

Main results 16

Socio-demographic factors related to incomplete childhood immunization (Table 2, page 22)

Variables	Category	Crude OR (95% CI)	P-value
Maternal Age (In years)	≤ 19	8.27 (5.19, 13.18)	< 0.001*
	>19	1	
Occupational status	Housewife	0.92 (0.61, 1.39)	0.71
	Farmer	1	
	Merchant	0.80 (0.41, 1.57)	0.52
	Other	1.43 (0.78, 2.62)	0.24
Monthly income (In ETB)	≤ 500	1	
	500 - 1000	0.67 (0.43, 1.03)	0.07
	≥ 1000	0.89 (0.50, 1.55)	0.68
Mother's educational status	Illiterate	1	
	Read and write	1.10 (0.53, 2.25)	0.79
	Elementary	1.01 (0.64, 1.58)	0.95
	Secondary and above	0.71 (0.37, 1.37)	0.31
Father's educational status	Illiterate	1	
	Read and write	1.21 (0.70, 2.09)	0.48
	Elementary	1.17 (0.74, 1.85)	0.48
	Secondary and above	1.10 (0.67, 1.81)	0.68
Residence	Rural	1	
	Urban	1.04 (0.60, 1.81)	0.86
Family size	≤ 5	0.45 (0.30, 0.65)	< 0.001*
	> 5	1	
Child sex	Male	1	
	Female	0.87 (0.60, 1.24)	0.46
Birth order	1	1	
	2 - 4	3.57 (2.08, 6.13)	< 0.001*
	≥ 5	4.49 (2.54, 7.94)	< 0.001*
Birth interval (In Months)	≤ 23	1.18 (0.75, 1.86)	0.4
	24 – 47	1	
	> 47	0.83 (0.44, 0.57)	0.5

*Statistically significant at $p < 0.05$

Mother's knowledge and attitude about immunization (Table 3, page 24)

Variables	Category	Crude OR (95% CI)	P-value
Knew about immunization	Yes<br>No	1<br>2.13 (1.46, 3.09)	 $< 0.001^{*}$
Knew the schedule of vaccines	Yes<br>No	1<br>1.06 (0.67, 1.67)	 0.8
Knew the benefits of immunization	Yes<br>No	1<br>6.30 (2.32, 17.08)	 $< 0.001^{*}$
Knew about vaccine side effects	Yes<br>No	1.49 (0.85, 2.56)<br>1	0.16
Attitude towards benefits of immunization	Positive<br>Negative	1<br>1.54 (0.63, 3.73)	 0.33
Perception about vaccine side effects	Positive<br>Negative	1<br>1.28 (0.86, 1.88)	 0.21
Attitude towards the last received child immunization	Positive<br>Negative	1<br>0.97 (0.68, 1.39)	 0.8

*Statistically significant at $p < 0.05$

Factors related to immunization service delivery (Table 4, page 25)

Variables	Category	Crude OR (95% CI)	P-value
Advised to vaccinate their child during their health institution visit	Yes<br>No	1<br>1.44 (0.62, 3.37)	 0.39
Information was received about the next vaccine schedule	Yes<br>No	1<br>1.81 (0.69, 4.79)	 0.22
Availability of immunization clinic	Yes<br>No	1<br>0.97 (0.62, 1.51)	 0.91
Difficulty of getting immunization shots	Yes<br>No	1.05 (0.68, 1.63)<br>1	0.79
Convenient hours for immunization	Yes<br>No	1<br>0.70 (0.48, 1.04)	 0.08
Postponing of the immunization schedule	Yes<br>No	1.89 (1.27, 2.81)<br>1	0.001^{*}

*Statistically significant at $p < 0.05$

Predictors of incomplete childhood immunization status (Table 5, page 26)

Variables	Category	Adjusted OR (95% CI)	P-value
Maternal Age (In years)	≤ 19	9.54 (5.03,18.09)	< 0.001*
	> 19	1	
Monthly income (In ETB)	≤ 500	1	
	500 - 1000	0.49 (0.23, 1.05)	0.07
	> 1000	1.59 (0.65, 3.85)	0.30
Family size	≤ 5	0.70 (0.34, 1.45)	0.34
	> 5	1	
Birth order	1	1	
	2 - 4	3.64 (1.63, 8.14)	< 0.001*
	≥ 5	5.27 (2.20, 12.64)	0.002*
Knew the benefits of immunization	Yes	1	
	No	5.51 (1.52, 19.94)	0.009*
Knew about vaccine side effects	Yes	0.96 (0.46, 2.01)	0.90
	No	1	
Perception about vaccine side effects	Positive	1	
	Negative	1.92 (1.01, 3.70)	0.05*
Information was received about the next vaccine schedule	Yes	1	
	No	0.20 (0.01, 2.45)	0.21
Convenient hours for immunization	Yes	1	
	No	1.06 (0.55, 2.04)	0.80
Postponing of the immunization schedule	Yes	1.30 (0.70, 2.41)	0.39
	No	1	

*Statistically significant at $p < 0.05$

Other analyses	17	Co-linearity diagnostic tests
**Discussion**		
Key results	18	The study assessed different factors and the variables which showed a significant association with incomplete immunization status; were maternal age, birth order, knowledge about immunization benefits and perception of vaccine side effects. (page 10)<br>Unavailability of vaccines on appointed immunization dates and migration of mothers were also indicated as key reasons for incomplete childhood immunization by the qualitative method. (page 11)
Limitations	19	Recall bias where mothers might not remember past histories for some immunization service delivery related questions.

Interpretation	20	In order to identify the EPI performance problems, still understanding the local challenges of immunization programs is of great importance.
Generalisability	21	The study results can be generalised to other Ethiopian districts with similar socio-demographic characteristics.
**Other information**		
Funding	22	The study was supported by Jimma University.

*Give information separately for cases and controls

Note: An Explanation and Elaboration article discusses each checklist item and gives methodological background and published examples of transparent reporting. The STROBE checklist is best used in conjunction with this article (freely available on the Web sites of PLoS Medicine at http://www.plosmedicine.org/, Annals of Internal Medicine at http://www.annals.org/, and Epidemiology at http://www.epidem.com/). Information on the STROBE Initiative is available at http://www.strobe-statement.org.
