# Supplementary material for: Factors associated with incomplete childhood immunization in Arbegona district, southern Ethiopia: a case – control study
Source: BMC Public Health. 2016 Jan 12;16:27. doi: 10.1186/s12889-015-2678-1 (PMC4711011; doi:10.1186/s12889-015-2678-1)
Supplement: Supplementary file 2 — Structured questionnaire for mothers/caregivers. (PDF 156 kb) [file 12889_2015_2678_MOESM2_ESM.pdf]

Structured questionnaire for mothers/caregivers

Informed Consent

Good morning/Good afternoon. I am _____________ who is a member of team conducting a study on determinant factors of childhood immunization defaulting in Arbegona district.

The purpose of this study is to find the reasons why eligible children, in this and other kebeles in the district, sometimes do not receive all the vaccines and find ways to address the challenges. Of the mothers who have children between ages of 12-23 months, you are selected randomly as one of the mothers/caregivers to participate in this study. The study will be conducted through interviews. The interview involves different questions regarding child immunization. We are asking you for a little of your time, about forty five minutes, to help in this study. We would like to assure you that confidentiality will be strictly secured throughout the study. All your information will be numbered and your name will not be used. Your answers to any of the questions will not be given to anyone else and no reports of the study will ever identify you. If a report of results is published, only information about the total group will appear.

The interview is voluntary. Your participation/ non-participation, or refusal to answer questions will have no effect now or in the future on services that you or any member of your family may receive from health service providers. If we should come to any question you don't want to answer, just let me know and I will go on to the next question; or you can stop the interview at any time. However, your honest answers to these questions will help us in better understanding of the factors related to incomplete childhood immunization in the district and will eventually help to design appropriate immunization services to children less than one year.

Are you willing to participate in this study? [ ] Yes [ ] No

Identification

District---------------------- Kebele-----------House No. /Gote --------- Serial no…………

Date of interview *(dd/mm/yyyy)* ------- /----------/ ---------------

The interviewee could be mother or the immediate caretaker

I. SOCIO-DEMOGRAPHIC CHARACTERISTICS

S.No	Questions	Responses	Skip pattern	Code
101	Who is the immediate caretaker of the child?	1. Mother<br>2. Father<br>3. Sister<br>4. Relatives<br>5. Other (specify)…………		
102	Age of the immediate care taker	………………years		
103	Marital Status of the immediate care taker	1. Married<br>2. Single<br>3. Widowed<br>4. Divorced<br>5. Separated<br>6. Other (specify)…………		
104	Ethnicity	1. Sidama<br>2. Amhara<br>3. Gurage<br>4. Oromo<br>5. Other (specify)…………		
105	Religion	1. Protestant<br>2. Orthodox<br>3. Muslim<br>4. Catholic<br>5. Other (specify)………		

106	Occupational Status	1. House wife<br>2. Daily labourer<br>3. Farmer<br>4. Merchant<br>5. Self employ<br>6. Government Employee<br>7. Other (specify)………		
107	Monthly income in birr	……………………..		
108	Educational Status	1. Illiterate<br>2. Read and write<br>3. Elementary<br>4. Junior secondary school<br>5. Secondary and certificate<br>6. College and above<br>7. Other (specify)………..		
109	Father's educational status	1. Illiterate<br>2. Read and write<br>3. Elementary<br>4. Junior secondary school<br>5. Secondary and certificate<br>6. College and above<br>7. Other (specify)………..		
110	Residence	1. Urban<br>2. Rural		
111	Family size	………………….		
112	Is there death of Child less than 5 years in the last one year?	1. Yes<br>2. No-------------------→	Q 115	
113	How many children less than five years died?	………………………		

114	Age of the child under investigation(In months)	……………………….		
115	Sex of the child under study	1. Male<br>2. Female		
116	Birth interval between the index child and the preceding child *(if the child had little brother /sister)?*	____________months		
117	Birth interval between the index child and the next child *(if the child had older brother /sister)*	_________months		
118	Birth order of the child *(if there are other children)*	_________		
**II. KNOWLEDGE AND ATTITUDE REGARDING CHILD IMMUNIZATION AND COMPLETION OF VACCINE SCHEDULES**				
201	Do you know about Vaccination?	1. Yes<br>2. No-------------------→	Q 211	
202	Which type of vaccination do you know?	1. BCG<br>2. Polio<br>3. Pentavalent<br>4. Measles<br>5. Other (specify)………..		
203	Which diseases can vaccines prevent?	1. Tuberculosis<br>2. Polio<br>3. Diphtheria<br>4. Pertussis<br>5. Tetanus		

		6. Measles<br>7. Hepatitis B<br>8. Haemophilus influenza B<br>9. Others (specify)…………		
204	Do you know the schedule of child immunization?	1. Yes<br>2. No-------------------→	Q 206	
205	When to immunize the child with the following vaccines? *(in weeks after birth)*	1. BCG …………………<br>2. Polio ………………….<br>3. Pentavalent……………<br>4. Measles ………………		
206	How do you know whether or not your child completed the immunization schedule?	1. I refer to immunization card<br>2. I follow health professional's/HEW's instructions<br>3. I refer to child's age<br>4. If child looks healthy<br>5. After measles vaccine<br>6. I don't know<br>7. Other (specify)…………		
207	Does vaccination has a benefit for the child?	1. Yes<br>2. No-------------------→	Q 209	
208	What are the benefits of immunization?	1. Immunity for the child<br>2. Prevent diseases<br>3. Control epidemic<br>4. Other ………………...		
209	Does immunization has side reaction?	1. Yes<br>2. No -------------------→	Q 211	

210	What is the side reaction of immunization?	1. Fever<br>2. Shivering<br>3. Pain<br>4. Vomiting<br>5. Other (specify)…………		
211	Giving immunization shots to children can prevent a disease	1. Strongly disagree<br>2. Disagree<br>3. Neutral<br>4. Agree<br>5. Strongly agree.		
212	Immunization shots are not safe for children.	1. Strongly disagree<br>2. Disagree<br>3. Neutral<br>4. Agree<br>5. Strongly agree.		
213	The staff at the clinic where you got your child's immunization shots was helpful.	1. Strongly disagree<br>2. Disagree<br>3. Neutral<br>4. Agree<br>5. Strongly agree.		
214	You normally had to wait for only a few minutes in the clinic when you took your child to get his/her immunization shots.	1. Strongly disagree<br>2. Disagree<br>3. Neutral<br>4. Agree<br>5. Strongly agree.		
215	Finding enough information about immunization shots that your child received was a problem for you.	1. Strongly disagree<br>2. Disagree<br>3. Neutral<br>4. Agree<br>5. Strongly agree.		

216	You had difficulty of finding out where immunization shots were available.	1. Strongly disagree<br>2. Disagree<br>3. Neutral<br>4. Agree<br>5. Strongly agree.		

**III. FACTORS RELATED TO IMMUNIZATION SERVICE DELIVERY**				
301	Have you ever taken your child to health institution for other services?	1. yes<br>2. No-------------------→	Q 307	
302	Why did you taken him/her to health institution?	1. For Growth Monitoring<br>2. Follow up for chronic care<br>3. Became sick<br>4. For check up<br>5. Other, specify---------------		
303	Did you informed or advised to vaccinate the child during your visit?	1. Yes<br>2. No-------------------→	Q 307	
304	Have you vaccinated your child?	1. Yes<br>2. No-------------------→	Q 306	
305	Which vaccine your child vaccinated?<br>(circle for the antigen provided during the health institution visit for other services)	1. BCG<br>2. Polio 1 2 3<br>3. Penta 1 2 3<br>4. Measles		

306	Why didn't you vaccinate your child?	1. Severely sick<br>2. Not good to vaccinate during illness<br>3. Other, Specify………..		
307	Where did you deliver the child?<br>**(Q 307- 312Asked if the respondent is the mother)**	1. Home<br>2. Health institution<br>3. Other, specify___		
308	Who attended the delivery?	1. Health professional<br>2. TBA<br>3. Lay person<br>4. Other, specify____		
309	Did you advised to vaccinate your child after delivery?	1. Yes<br>2. No		
310	Did you attend post-natal care after delivery of the child?	1. Yes<br>2. No		
311	How many times did you attend post natal care?	………………………		
312	Did you advised to vaccinate your child at post natal period?	1. Yes<br>2. No		
313	The staff at the clinic always informed you about when the next immunization shot was due?	1. Yes<br>2. No		

314	Are clinics providing immunization shots available in your area?	1. Yes<br>2. No		
315	Is it difficult to get the immunization shots?	1. Yes<br>2. No		
316	The immunization clinics that you visited had hours that were convenient for you?	1. Yes<br>2. No		
317	Was there any occasion in which you returned home without getting vaccination during your appointment?	1. Yes<br>2. No		
318	If yes, what was the reason for not getting vaccination?	1. Vaccine not available<br>2. Vaccinators were absent<br>3. I Don't know<br>4. Other (specify)………..		

Thank you for your cooperation!

Name of the Interviewer--------------------------- Signature ----------------------------
