# Supplementary material for: Factors associated with incomplete childhood immunization in Arbegona district, southern Ethiopia: a case – control study
Source: BMC Public Health. 2016 Jan 12;16:27. doi: 10.1186/s12889-015-2678-1 (PMC4711011; doi:10.1186/s12889-015-2678-1)
Supplement: Supplementary file 3 — Focus Group Discussion guide. (PDF 78 kb) [file 12889_2015_2678_MOESM3_ESM.pdf]

Focus Group Discussion guide

Thanks for coming. My name is………………. and I am working a research on factors that determine childhood immunization defaulting. Your presence is very important. We are going to have a group discussion. I will ask you very general questions.

I want to learn from your experiences about child immunization. I am interested in all your ideas, comments and suggestions. There are no rights or wrong answers. All comments, both positive and negative, are welcome. Please feel free to disagree with one another; we would like to have many points of views. Whatever you say will not make me feel good or bad or affect me in any way. So feel free to give frank and honest answers.

If you don't mind, I will record (audiotape) the discussion. The purpose is to ensure that I don't miss anything you said. All comments are confidential, used for research purpose only.

I want this to be a group discussion. So you need not wait for me to call on you. Please speak one at a time, so that the tape-recorder can pick up everything. You have to respect the views of others even if it may be different from yours. We have a lot of ground to cover, so I may change the subject or move ahead. Please stop me if you want to add something. You all participants have an obligation to keep confidential what you hear from other participants.

Points for Focus Group Discussion for Health Professionals

1. What do you think about vaccination and its importance?
2. How is the vaccination acceptability in the community?
3. What problems are there in the immunization service delivery?
4. Is the immunization service integrated with other services?
5. Why do you think mothers don't complete their child's immunization schedule?
6. Do you think gaps from health professionals exist that contribute for the incomplete immunization status of children? What are these gaps?
7. What do you think the health professionals need to do to improve the immunization service delivery?
8. What needs to be improved, generally, to increase the number of fully vaccinated children?

Points for Focus Group Discussion for Health Extension Workers

1. What do you think about vaccination and its importance?
2. How is the vaccination acceptability in the community?
3. How do you mobilize mothers to go to immunization services?
4. Is there defaulter tracing system?
5. What are your responses to mothers who visit outreach units for immunization without immunization card or any problem relating to immunization?
6. Why do you think mothers don't complete their child's immunization schedule?
7. Is there any problem in the community that inhibited immunization completion of the children? If then what are the problems?
8. Are you motivated enough to cover areas/communities due for immunization? Why?

9. Is the support given from supervisors and Health Office staffs adequate?

10. What are the possible solutions to increase the number of fully vaccinated children?
