# Supplementary material for: Factors associated with incomplete childhood immunization in Arbegona district, southern Ethiopia: a case – control study
Source: BMC Public Health. 2016 Jan 12;16:27. doi: 10.1186/s12889-015-2678-1 (PMC4711011; doi:10.1186/s12889-015-2678-1)
Supplement: Supplementary file 4 — In-depth interview guide. (PDF 79 kb) [file 12889_2015_2678_MOESM4_ESM.pdf]

In-depth interview guide

Thanks for your will. My name is………………. I am working a research on factors that determine childhood immunization defaulting. I am going to have an in-depth interview for the next 45 minutes.

I am interested in all your ideas, comments and suggestions. There are no rights or wrong answers. All comments, both positive and negative, are welcome. So feel free to give frank and honest answers.

If you don’t mind, I will record (audiotape) the discussion. The purpose is to ensure I don’t miss anything you said. All comments are confidential, used for research purpose only. I have a lot of ground to cover, so I may change the subject or move ahead. Please stop me if you want to add something.

Points for In-depth Interview with Head of the District Health Office, Disease Prevention and Health Promotion core process coordinator; and Health Extension Supervisors.

1. How is the EPI program being managed?
2. What problems exist in the implementation of the EPI program?
3. Are there enough resources for the program? If no, which resources are not fulfilled?
4. Was there occasions in which vaccine shortage was encountered in last year?
5. How you identify and approach your target population?
6. Is dropout rate monitored monthly and feedback given to Health Extension Workers?
7. Do you have adequate staff to cover the population target for the immunization schedule?
8. Is there adequate logistic for supervisory activities?
9. Do you supervise all the sub-districts with a plan schedule? If no, why?
10. Do you always meet HEWs at post during your visit?
11. In your opinion, what are the most challenging issues during your visits to the Sub-districts?
12. What problems do you think favour incomplete immunization status of children?

Points for In-depth Interview with Heads of Health Centres

1. Is the EPI activity managed by trained personnel?
2. Is the EPI service integrated with other services? How?
3. Why do you think eligible children missed for immunization during their health facility visits?
4. Do you evaluate your EPI service performance regularly? How?
5. Are there problems that affect the immunization service delivery? What are they?
6. How do you tried to solve these problems?
7. Why do you think mothers do not complete their child immunization schedule?
8. What gaps, related to the immunization service delivery, do you think contribute for incomplete immunization status of children?
